# Changes in e-cigarette use and related behaviors following the 2022 e-cigarette tax increase in China: A prospective longitudinal observational study

**DOI:** 10.18332/tid/208848

**Published:** 2025-12-04

**Authors:** Tingzhong Yang, Sihui Peng, Randall R. Cottrell

**Affiliations:** 1 Maternal and Child Healthcare Service Department, Yongkang Women and Children's Health Hospital, Yongkang, China; 2 Center for Tobacco Control Research, School of Medicine, Zhejiang University, Hangzhou, China; 3 Injury Control Research Center, West Virginia University, Morgantown, United States; 4 School of Medicine, Jinan University, Guangzhou, China; 5 School of Health and Applied Human Sciences, University of North Carolina, Wilmington, United States

**Keywords:** electronic cigarettes, e-cigarette tax, e-cigarette advertising exposure

## Abstract

**INTRODUCTION:**

Although the relationship between taxation and conventional cigarette use is well established, little is known about the association with electronic cigarette (e-cigarette) use and no study has accounted for the potential delayed effects of e-cigarette policies beyond the observation period. This study aimed to evaluate the impact of China’s 2022 e-cigarette tax increase on e-cigarette use.

**METHODS:**

A prospective longitudinal observational design was utilized by collecting three-waves of online survey data in China. Participants were recruited through social media platforms. Baseline data were collected in October 2022, with follow-up survey conducted in March and August 2023. Participants were eligible if they were aged ≥18 years, reported using e-cigarettes in the past 30 days at baseline, and provided informed consent to be recontacted for follow-up. The Friedman test and the Cochran's Q test were used to assess differences across the waves. The Wilcoxon signed-rank test and the Tukey test were used to make pairwise comparisons between the groups.

**RESULTS:**

There were 116 participants recruited at baseline and 91 (78.5%) of them complete all three surveys. E-cigarette use (ECU) decreased by 33.0% (95% CI: 23.7–42.3) in wave 2 after the tax increase was implemented, and by an additional 35.2% (95% CI: 26.8–43.7) in wave 3. The overall quit rate was 68.2% (95% CI: 60.3–75.9). However, 74.2% (95% CI: 65.9–82.5) of e-cigarette users whose quit switched to conventional cigarettes, resulting in an absolute cessation prevalence of only 17.6% (95% CI: 7.8–25.4). While perceived risk for ECU (χ^2^=0.41, p>0.05), perceived severity for ECU (χ^2^=1.02, p>0.05), and behavioral beliefs (χ^2^=2.28, p>0.05) did not change following the e-cigarette tax increase. Friends' attitudes (χ^2^=8.74, p<0.05), coworkers' attitudes (χ^2^=4.71, p<0.05), and exposure to e-cigarette advertising (Q=9.76, p<0.01) showed significant decreases.

**CONCLUSIONS:**

This study suggests a possible association between the China’s 2022 e-cigarette tax increase and changes in e-cigarette use. However, its effectiveness was diminished due to the large number of e-cigarette users who switched to conventional cigarettes. China's 2022 e-cigarette tax increase also affected social norms regarding e-cigarette use and exposure to e-cigarette advertising. The findings may inform future policy considerations for a comprehensive strategy.

## INTRODUCTION

Global use of electronic cigarettes (e-cigarettes) has been expanding, encompassing various facets of society, economics, health, and behaviors. Taxing e-cigarette use has surfaced as a pivotal strategy for controlling e-cigarette use and mitigating associated health risks^1^. While numerous studies have explored the influence of conventional cigarette taxes on smoking behavior^2-4^, there is limited research examining the impact of e-cigarette taxes on e-cigarette use^5^. In October 2022, the Chinese Ministry of Finance, the General Administration of Customs, and the State Administration of Taxation collectively released Announcement No. 33. This transitioned e-cigarettes from being taxed as regular value-added goods to being included within the cigarette consumption tax bracket^6^. The Announcement specifies that the tax rate for the production (or import) of e-cigarettes is set at 36%, which aligns with the rate for Class B conventional cigarettes, the rank second price in tobacco industry system in China. Before this regulation, e-cigarettes were only subject to a value-added tax of 13%. Furthermore, the wholesale tax rate for e-cigarettes was established at 11%, mirroring the rate for all conventional cigarettes. The Announcement was put into effect on 1 November 2022. When the policy was implemented, the incremental tax burden ranged from 8.2 to 14.2 RMB (1000 Chinese Renminbi about US$140) per cartridge^7^. This is signifying that the maximum tax burden could potentially rise to over three times the previous tax amount. Following the tax hike, the excise tax now constitutes approximately 30% of the e-cigarettes price in China. In less than three weeks, following the enforcement of the consumption tax on e-cigarettes, there was a noticeable increase in the prices of products such as Relx and Pomelo^8^.

This study aims to examine the impact of the 2022 e-cigarette tax hike on e-cigarette use in China. Compared to previous studies, this study has several advantages. Firstly, previous studies assessing the impact of tax policies on e-cigarette use were primarily cross-sectional studies^9-11^. This research employs a prospective longitudinal observational design, which will enhance our ability to establish a possible causal relationship and further understanding of the policy impact on e-cigarette use. Secondly, the influence of public policies on people’s behavior is not necessarily immediate^12^. A longitudinal study has typically been limited to a single-observation time point post-policy implementation^13^. These studies often do not account for the potential delayed effects of these policies beyond the observation period. Such an approach may not fully capture the real-world impact of public policies. Our study will carry out observations in multiple time points. Thirdly, existing studies on the effectiveness of increasing e-cigarette taxes involved only a single behavior variable related to e-cigarette use^14,15^. This study will incorporate multiple variables associated with e-cigarette use.

The Individual Behavior Cognition and Social Influence (BCSI) theory, suggests that policies increasing e-cigarette taxes can lead to changes beyond just e-cigarette usage behavior^16^. They can also affect other aspects related to e-cigarette use, including behavior cognition, social norm influence, and social environment. These may serve as direct variables of policy impact or intermediate variables between policy and e-cigarette use behavior^12,16^. If a policy has the potential to influence these multi-dimensional variables, the probability of it affecting behavioral change is more robust than if it could only impact a single behavioral aspect. Our hypothesis posits that the policy of increasing e-cigarette taxes will not only decrease e-cigarette use behavior, it will also reshape the social climate surrounding tobacco control, thereby prompting alterations in other factors associated with e-cigarette use.

The core belief variables in the BCSI theory, such as behavioral belief, perceived risk, perceived severity, and social norms, are well-established constructs that align closely with those found in the Theory of Reasoned Action (TRA) and the Health Belief Model (HBM)^16,17^. According to the TRA, both attitudes toward the behavior and the social norms shape individual’s behavior. Similarly, the HBM posits that health-related behaviors are primarily influenced by individuals’ perceptions of risk and the severity of potential consequences. These theoretical overlaps highlight the conceptual alignment between the BCSI framework and these foundational models in health behavior research. Some studies have found these variables are positively associated with e-cigarette use^18,19^. They are, however, challenging to influence and modify. Nonetheless, it is interesting to examine the spillover effect of the tax policy, and specifically whether the increase in e-cigarette tax influences people’s cognition on e-cigarette use by impacting the overall atmosphere of tobacco control.

The social environment serves as another variable within the BCSI theory. Previous study has identified a positive correlation between exposure to e-cigarette advertising and e-cigarette use^20^. This study will examine whether the policy of increasing e-cigarette taxes influences environmental e-cigarette advertising. If the correlation is established, it would provide an additional avenue to understand the relationship between e-cigarette advertising and e-cigarette use. Consequently, we have incorporated e-cigarette advertising exposure into our research framework.

Some studies have also found that e-cigarette tax increases may lead to a decrease in e-cigarette use^5,21^. However, they have also been associated with an increase in conventional cigarette use and a reduction in cessation of conventional cigarette use. This suggests that e-cigarettes and conventional cigarettes may serve as substitutes for one another^22,23^. Therefore, conventional cigarette use is also being examined in this study. Given the unique characteristics of the current Chinese tobacco market, exploring the relationship between e-cigarette and conventional cigarette use holds significant value.

## METHODS

### Data source

A prospective longitudinal observational study was devised to examine the temporal trends and changes in e-cigarette use, perceptions of e-cigarette use, social norms, and exposure to e-cigarette advertising before and after the implementing e-cigarette tax increase policy. The observation included three waves. Wave 1, administered in October 2022, occurred before the e-cigarette tax increase policy was implemented; wave 2, administered March 2023, and wave 3, administered August 2023, took place after the policy was implemented. The entire observational period encompassed ten months. Effects with a time lag are a frequent occurrence in public policy work^24,25^. It could take time to observe the impact of a new policy given the complex nature of policy enforcement. Thus, our assessment of policy effectiveness was not conducted immediately after the policy’s implementation but rather after allowing sufficient time for the tax increase to impact the market. Consequently, our second wave was conducted post-implementation, which differs from the approach taken in previous studies^3^.

### Data collection

Participants were recruited via an advertisement posted on WeChat, which is one of the most popular social media platforms in China. Inclusion criteria were users who: 1) were aged ≥18 years, 2) were proficient in the Chinese language, and 3) were willing to provide follow-up information at three scheduled observation points. Participants were excluded if they refused to provide this information or had a medical condition that could limit or preclude their participation. Within the registration system, potential participants were screened to ascertain eligibility. Upon consent with an electronic informed consent letter, participants received electronic instructions on how to proceed. After reading the consent letter, they were asked to provide an e-consent by tapping the ‘Confirmation and Authorization’ button at which point they were then directed to the questionnaire. A special administrative WeChat group was established to manage the baseline and follow-up data collection, using a unique QR code for each respondent^26^.

The investigation was managed by Wenjuanxing, a survey service platform similar to SurveyMonkey. The online questionnaire link was posted to the participant WeChat group. The same survey protocol was used for each wave to assure homogeneity of data administration and collection. As appropriate, a token of appreciation, 50 RMB (about US$7) was given to those participants who completed all three questionnaires. All responses were anonymous. The authorized screen name and a self-determined code were obtained as the unique identifier for each participant.

### Measurements

*Demographic characteristics*

Individual demographic characteristics were recorded including age, gender, ethnicity, education level, marital status, occupation, and family annual income.

*E-cigarette use status definitions*

Adult use of e-cigarettes was defined as using e-cigarettes in the past 30 days^27^, and measured by the question: ‘In the past 30 days, have you used e-cigarettes?’. The frequency of e-cigarette use refers to the number of times e-cigarettes were used each day. The intensity of e-cigarette use refers to the length of time per e-cigarette use. Longer use time was categorized as ≥10 minutes. Those who tried to quit e-cigarettes are defined as individuals who experience more than three attempts to quit the use of e-cigarettes, each attempt lasting for more than three days.

*Conventional cigarettes use*

The standard measure of conventional cigarette use recommended by the WHO^27^ was used. Current smoker was defined as someone who smoked cigarettes at the time of the survey. They were asked whether they currently smoked (use conventional cigarettes), and response options were ‘yes’or ‘no’.

*Behavior belief on e-cigarette use*

This was measured by the question: ‘Is e-cigarette use a bad habit?’. Responses were on a 5-point Likert scale ranging from ‘strongly disagree’ to ‘strongly agree’.

*Perceived risk of e-cigarette use*

This was measured by the question: ‘Is there a possibility of getting sick because of e-cigarette use?’. Responses were on a 5-point Likert scale ranging from ‘not possible’ to ‘very possible.’

*Perceived severity of e-cigarette use*

This was measured by the question: ‘Would it be serious if you got sick from e-cigarette use?’. Responses were on a 5-point Likert scale ranging from ‘not serious’ to ‘very serious’.

*Exposure to e-cigarette advertising*

Participants were asked whether they had seen any e-cigarette advertisements in the last six months. Responses to this item were recorded as ‘no’ or ‘yes’.

*Attitudes towards e-cigarette use among friends, coworkers, and family members*

This was measured with the question: ‘What are your [subjects'] attitudes toward e-cigarette use?’. The subjects included 'friends', 'coworkers', and 'family members', which were present solely in one question. Responses were on a 5-point Likert scale ranging from ‘not supportive’ to ‘very supportive’.

### Data analysis

All data were saved as Microsoft Excel files and imported in to SAS (9.4 version) for statistical analysis. Continuous variables were not normally distributed as determined by normality testing; therefore, non-parametric testing methods were utilized to conduct the analysis. The Friedman test was used to examine differences across the three waves. For categorical variables, differences across the three waves were assessed using non-parametric tests for repeated measures. Specifically, for binary variables, the Cochran's Q test was used, and the Q statistic is reported^28,29^. Pairwise comparisons between the groups were performed using *post hoc* comparison methods. The Wilcoxon signed-rank test was applied for the former, while the Tukey test was used for the latter. A two-tailed alpha level of ≤0.05 was considered statistically significant.

## RESULTS

**Figure 1:**
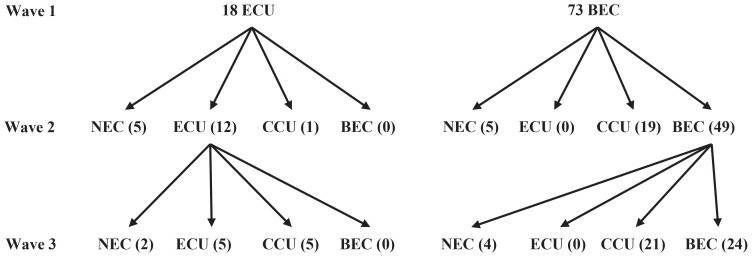
Changes in e-cigarette users during the three observational waves from 2022 to 2023 (N=91)

A total of 116 participants completed the survey at baseline, 106 of them completed the second survey, and 91 (78.5%) completed all three surveys. Of the respondents, 39.6% were female, 48.8% were aged <25 years, 78.0% were unmarried, and almost 95% had some college level training (Table 1). Of the 91 participants who completed all three surveys, 18 (19.8%) were individuals who only used e-cigarettes (ECU) and 73 (80.2%) were individuals who used both e-cigarettes and conventional cigarettes (BEC). Figure 1 shows the change process of both ECU and BEC from wave 1 to wave 3.

**Table 1:** The characteristics of the participants who completed three-wave longitudinal observational survey from 2022 to 2023 (N=91)

*Characteristics*	*n*	*%*
**Age** (years)		
<20	3	3.3
20–24	41	45.5
25–29	15	16.5
30–34	18	19.5
≥35	14	15.4
**Gender**		
Male	55	60.4
Female	36	39.6
**Ethnicity**		
Han	80	87.9
Minority	11	12.1
**Marital status**		
Unmarried	71	78.0
Married	18	19.8
Divorced	2	2.2
**Education level**		
High school and lower	5	5.4
Junior college	27	29.7
College and higher	59	64.8
**Occupation**		
Administration, commercial and service	17	18.7
Science and education	34	37.4
Technology	13	14.2
Student	27	29.7
Unemployed	2	2.2
**Family annual income** (RMB)		
<20000	7	7.7
20000–39999	11	12.1
40000–59999	12	13.2
60000–79999	12	13.2
80000–99999	10	11.0
≥100000	39	42.9

RMB: 1000 Chinese Renminbi about US$140.

Among the 18 ECU from wave 1 to wave 2, 12 (67%) continued to use e-cigarettes while 5 (28%) of them used neither e-cigarettes nor conventional cigarettes (NEC), and 1 (6%) transitioned to conventional cigarette use (CCU). When the third survey was administered, of 12 ECU individuals in wave 2, only 2 (11%) had become NEC individuals, 5 (28%) transitioned to CCU, and 5 (28%) continued with ECU. Throughout the entire observation period, 7 (38%) of the former e-cigarette users quit all cigarette use. The e-cigarette cessation rate was 38.9% (95% CI: 16.4–61.0), and the rate for those switching regular cigarettes was 33.3% (95% CI: 21.1–54.5). In total, 72.2% (95% CI: 52.7–91.7) of e-cigarette users either quit or switched to conventional cigarettes.

Among 73 BEC individuals from wave 1 to wave 2, only 5 (7%) of them became NEC individuals, 19 (23%) transitioned to CCU, and 49 (67%) continued with BEC. At wave 3, of the 49 BEC individuals in wave 2, only 4 (8%) became NEC individuals, and 21 (43%) transitioned to CCU, while 24 (49%) continued with BEC in wave 3. Throughout the entire observation period, the prevalence of e-cigarette cessation was 12.3% (95% CI: 5.0–19.6) and the prevalence for switching to CCU was 54.8% (95% CI: 42.8–66.1). In total, the crude e-cigarette cessation prevalence was 67.1% (95% CI: 60.2–74.1). It should be noted we did not find any cases of NEC or CCU individuals who transitioned to ECU in the observation period.

**Figure 2:**
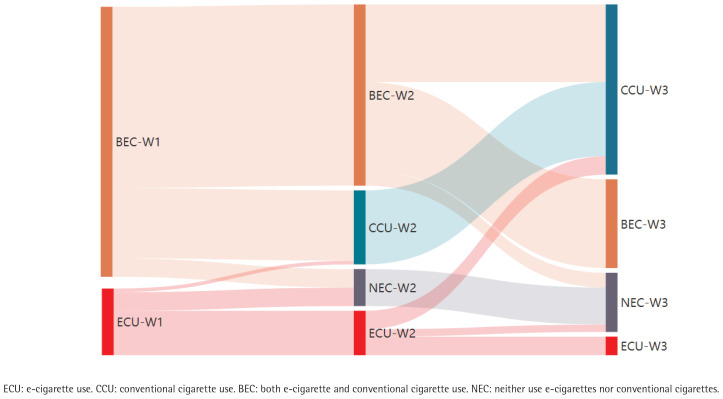
Sankey diagram of ECU, BEC, CCU and NEC during the three longitudinal observational waves 2022 to 2023 (N=91)

In wave 2, of the participants, 30 became e-cigarette quitters, and the crude cessation prevalence was 33.0% (95% CI: 23.7–42.3). In wave 3, of the participants, 32 became e-cigarette quitters, and the crude cessation prevalence was 35.2% (95% CI: 26.8–43.7). In whole observation period, 62 became e-cigarette quitters, and the cessation crude prevalence was 68.1% (95% CI: 60.3–75.9), 16 became NEC individuals and the prevalence was 17.6% (95% CI: 7.8–25.4), 46 transitioned to CCU and the prevalence was 50.6% (95% CI: 40.2–60.6), among 91 e-cigarette users (Figure 2).

Table 2 shows changes of e-cigarette use behavior and related variables with the e-cigarette tax increase. While there was no significant change from wave 1 to wave 3 in the cessation attempt among ECU individuals (Q=0.97, p>0.05), the actual cessation prevalence of ECU significantly increased (Q=72.36, p<0.01). Additionally, there was a significant increase in the switch from e-cigarette use to conventional cigarettes throughout the entire observation period (Q=9.70, p<0.01). While there was a significant decrease in the daily frequency of ECU (Q=39.33, p<0.01), there was also a significant increase in the intensity of ECU each with each wave (Q=17.66, p<0.01). The prevalence of exposure to e-cigarette advertising before and after the implementation of the e-cigarette tax policy was significantly decreased (χ^2^=9.76, p<0.01). There was no significant change in the average higher monthly cost prevalence among the three waves (χ^2^=1.36, p>0.05). The implementation of the e-cigarette tax policy did not result in changes in perceived risk for ECU (χ^2^=0.41, p>0.05), perceived severity for ECU (χ^2^=1.02, p>0.05), or behavior belief (χ^2^=2.28, p>0.05). There was a significant decrease in friends’ and coworkers’ attitudes towards e-cigarette use, χ^2^ values of 8.74 (p<0.05) and 4.71 (p<0.05) respectively, but no significant changes were observed in family members’ attitudes towards e-cigarette use (χ^2^=0.21, p>0.05).

**Table 2:** Before and later change of ECU related behavioral and perceived variables during the three-wave observational survey from 2022 to 2023 (N=91)

*Items*	*Wave 1* *%*	*Wave 2* *%*	*Wave 3* *%*	*χ^2^ (Q)*
ECU cessation prevalence^+^	0^a^	33.0^b^	35.2^b^	72.36^**^
ECU frequency at each day^+^	68.1^a^	68.9^a^	8.5^b^	39.33^**^
Higher intensity of ECU prevalence at each time^+^	7.7^a^	36.1^b^	32.2^b^	17.66^**^
ECU tried cessation prevalence^+^	61.6^a^	67.2^a^	62.7^a^	0. 97
Switching to conventional cigarettes prevalence^+^	19.3^a^	28.0^b^	25.6^b^	9.70^**^
Average higher monthly cost prevalence (>250 RMB) of e-cigarette users^+^	71.4^a^	65.6^a^	73.6^a^	1.36
Exposure to e-cigarette advertising^+^	69.2^a^	41.6^b^	46.2^b^	9.76^**^
	***Mean***	***Mean***	***Mean***	
Perceived risk for ECU^‡^	2.16^a^	2.11^a^	2.17^a^	0.41
Perceived severity for ECU^‡^	2.87^a^	2.95^a^	2.92^a^	1.02
Behavior belief on e-cigarette use^‡^	3.94^a^	3.29^a^	3.35^a^	2.28
Friends’ attitudes toward e-cigarette use^‡^	3.33^a^	3.09^b^	3.00^b^	8.74*
Coworkers’ attitudes toward e-cigarette use^‡^	3.22^a^	3.18^a^	3.00^b^	4.71*
Family members’ attitudes toward e-cigarette use^‡^	2.35^a^	2.37^a^	2.31^a^	0.21

ECU: e-cigarette use.

a,b The differences between observation groups at different time points. The same letter indicates no significant difference between groups, while different letters indicate a significant difference between groups.

+ Cochran's Q test.

‡ Friedman test.

* p<0.05;

** p<0.01.

## DISCUSSION

This study examined the effects of China’s 2022 electronic cigarette tax increase on ECU behavior. The findings revealed that the prevalence of ECU cessation was 33.0% five months after implementing the new tax policy, and 35.2% after ten months. These findings indicate that China’s 2022 electronic cigarette tax increase, where the excise tax constituted about 30% of the e-cigarette price, significantly influenced ECU behavior. An online survey by Minami and Teo^10^ revealed that an increase in e-cigarette prices led to not only an increase in the rates of e-cigarette users who would quit, but also a rise in the rates of conventional cigarette users who would increase smoking^10^. Pesko et al.^5^ found that a 10% increase in the prices of disposable e-cigarettes was associated with a decrease in e-cigarette use days among e-cigarette users by 9.7%. Moreover, it was associated with a 17.9% reduction in e-cigarette use days by the entire sample^5^. Given the varied methodologies employed in the aforementioned studies, it is not feasible to directly compare the extent of ECU reduction among them. However, it can be confirmed that China’s 2022 electronic cigarette tax policy significantly decreased ECU behavior.

It is important to note that the majority of those who quit e-cigarettes transitioned to conventional cigarettes. This study discovered that the rate of transitioning into CCU was 33.3% among e-cigarette users, and 46.2% among e-cigarette quitters. Of the BEC individuals, the CCU transitioning rate was 54.8%, and 81.6% among BEC quitters. Of ECU and BEC individuals, the rate was 50.6% for users, and 74.2% for quitters. Several studies have established that e-cigarettes and conventional cigarettes can serve as substitutes^11,22,23^. It is worth noting that a high prevalence of ECU quitting in response to an increase in e-cigarette taxes. However, the majority of these individuals transitioned to CCU, which significantly diminishes the impact of the policy. In fact, the absolute prevalence of ECU cessation was only 17.6%. Despite the retail tax on e-cigarettes being equivalent to that of conventional cigarettes in China following the e-cigarette tax increase, a significant disparity exists in their production costs. Consequently, the price of conventional cigarettes is considerably lower than that of e-cigarettes, leading to higher daily costs for e-cigarette users compared to conventional cigarette smokers. This has prompted a significant number of e-cigarette users to react to the surge in e-cigarette prices by transitioning to conventional smoking methods. The findings suggest that, to effectively influence overall smoking behavior, taxes on both conventional cigarettes and e-cigarettes should be increased simultaneously. In designing tax policies, it is important to avoid making one product more attractive or easier to choose simply due to its lower price. As the cost of e-cigarettes rises, some users, particularly younger individuals or those who are more price-sensitive, may switch to using conventional cigarettes. This substitution effect represents an unintended consequence of the tax policy, further illustrating the need to consider the interrelationship between different products when designing such policies. Price changes in both e-cigarettes and conventional cigarettes may not lead to the expected health benefits; instead, they could drive certain groups toward more harmful smoking methods, thereby worsening public health issues.

These results underscore the necessity of embedding e-cigarette taxation within a comprehensive tobacco control framework. Such a framework should include not only increased cigarette taxes but also enhanced smoking cessation support services and extensive public education campaigns, particularly targeting younger populations. The goal is to reduce price disparities and provide better support to effectively prevent consumers from switching to more harmful tobacco products.

The total prevalence of e-cigarette cessation reported in this study is a cumulative figure spanning several observation periods. This approach differs from previous studies that utilized one-time observations before and after policy implementation, potentially overlooking the delayed effects of the policy implementation^5,23^. Nevertheless, it remains undetermined what transpired during the third wave. It raises the question of whether the delayed effects of the e-cigarette tax increase policy will continue to impact people’s ECU after the final observations. Generally speaking, the effects of policy implementation cannot become apparent to many people immediately. For tax policies, especially those involving e-cigarette tax increases, changes in consumer behavior may take some time to fully materialize. For example, consumers may have stored some previous products and have not yet responded to the price increase of the new products. They need time to adapt to the new prices, assess the affordability of the products, and adjust their purchasing behaviors. Some studies found that cigarette excise tax increases had long-term effects (1989–1995) on consumer behavior, including a rise in the average levels of tar, nicotine, and carbon monoxide consumed per pack. This occurred as consumers substituted across tiers and brands, suggesting a long-term negative impact on health outcomes^30-32^. If so, what proportion of e-cigarette users might eventually quit? Further research is essential to answer these queries.

This study demonstrated that while the frequency of e-cigarette use declines with the decrease in e-cigarette tax, the intensity of each e-cigarettes usage session actually increases. This is attributed to the fact that people can more readily control the frequency of e-cigarette usage in response to policy pressure. However, once they start using, they tend to be governed by cravings^12^. This study revealed that while the prevalence of attempted ECU cessation did not significantly change, the actual prevalence of successful ECU cessation and the rate of switching to CCU among ECU quitters significantly increased in response to the e-cigarette tax increase. This suggests that the impact of China’s e-cigarette tax increase in 2022 on actual smoking cessation is substantial, which is consistent with other studies^9,33^. Monthly expense on tobacco products is also an evaluation indicator of tax policy. However, this study did not find any significant changes in the average cost of e-cigarette users with e-cigarette tax increase. This could be attributed to a combination of factors, such as the increase in ECU intensity and the decrease in frequency due to the e-cigarette tax increase.

This study found that behavioral beliefs, perceived risks, and severity concerning ECU remained unchanged in response to the e-cigarette tax increase. Moreover, the support attitudes towards e-cigarette use among friends and coworkers significantly decreased with the increase in e-cigarette tax, but family members’ attitudes remained unchanged. Prior research indicates that the impact of friends and colleagues’ norms surpasses that of family members^16^. This could potentially elucidate why policies affect friends and colleagues’ norms, but do not affect family members. Moreover, social norms have a wide-ranging influence on individuals’ actions and could potentially impact diverse facets of e-cigarette use behavior^12,17^.

This study found there was a significant decrease in reported e-cigarette advertising exposure after the e-cigarette tax increase was implemented. The direct reason for the decrease is that, with the introduction of the policy, the number of e-cigarette users has decreased. It is also possible that this could be contributed to by both changes in marketing strategies of the e-cigarette industry and consumer disengagement from e-cigarette producers.

### Limitations

There are several limitations to this study. First, sample attrition may introduce ‘cluster’ bias. This was a self-selected sample of volunteers who may not represent the behaviors of the general population. In this study, participants who dropped out before the final wave tended to have a lower level of education. No significant differences were found in terms of age, gender, ethnicity, occupation and family annual income. As with all longitudinal studies, those who remain in the study may be differ from those who dropped out, potentially affecting the generalizability of the findings. A more sophisticated design and more representative sample would be necessary to resolve this problem. Second, perceived beliefs were assessed with single-item question. In this study, the term ‘perceived beliefs’ refers specifically to perceived risk and severity, which is not an abstract concept and can be clearly defined and understood. Health behavior theory suggests that these variables be measured using a single-item question^12,17^. This approach has been widely applied in previous research^34,35^. However, further research also needs to minimize potential measurement bias. Thirdly, while the 10-month follow-up offers important short- to medium-term insights, it may not fully capture the long-term behavioral shifts and evolving social norms that could result from a national tax policy. A longer observation period would be beneficial to assess the sustainability and cumulative effects of the tax intervention. Future studies with extended follow-up are expected to better understanding of the full trajectory of policy impact. Fourthly, the sample size of this study was small, which may limit the statistical power to detect small or subgroup effects. However, the prospective longitudinal panel design, with repeated measures collected from the same participants, enhances the efficiency of statistical comparisons by reducing within-subject variability. In this study, we conducted *post hoc* power estimates using the Geisser-Greenhouse F test, based on a sample size of 91, a significance level of 0.05, and model-derived effect sizes. All variables demonstrated power levels above 0.80, which is considered acceptable^36^. While these estimates suggest adequate power for medium-to-large effects, we acknowledge that *post hoc* power analysis has methodological limitations and should be interpreted with caution. To improve the methodological rigor of future studies, estimating the sample size while considering additional influencing factors is recommended during the study planning phase.

## CONCLUSIONS

This study has contributed two important findings. Firstly, China’s 2022 e-cigarette tax policy has a notable positive impact on reducing e-cigarette use. However, this effect was potentially diminished by the lenient taxes in the conventional cigarette market, resulting in the majority of e-cigarette users switching to conventional cigarette use. Secondly, e-cigarette tax increases affected not only individual e-cigarette use behavior but also impacted social norms and perceived e-cigarette advertising exposure.

## Data Availability

The data supporting this research are available from the first author or corresponding author on reasonable request.
